# Efficacy and safety of pre-gastroscopy commercial carbohydrate-rich whey protein beverage vs. plain water: a randomised controlled trial

**DOI:** 10.1038/s41598-022-22363-1

**Published:** 2022-10-17

**Authors:** Bee Chen Lua, Mohd Nizam Md Hashim, Mung Seong Wong, Yeong Yeh Lee, Andee Dzulkarnaen Zakaria, Zaidi Zakaria, Wan Zainira Wan Zain, Syed Hassan Syed Abd Aziz, Maya Mazuwin Yahya, Michael Pak-Kai Wong

**Affiliations:** 1 School of Medical Sciences, Universiti Sains Malaysia, Kota Bharu, Kelantan Malaysia; 2 Department of Surgery, Hospital Universiti Sains Malaysia, Kota Bharu, Kelantan Malaysia; 3 Endoscopy Unit, Hospital Universiti Sains Malaysia, Kota Bharu, Kelantan Malaysia; 4 Department of Internal Medicine, Hospital Universiti Sains Malaysia, Kota Bharu, Kelantan Malaysia

**Keywords:** Medical research, Clinical trial design, Randomized controlled trials

## Abstract

Clinical benefits and safety of carbohydrate loading pre-gastroscopy remain unclear. We aimed to determine the effects of a commercial carbohydrate-rich whey protein beverage versus plain water given pre-gastroscopy on gastric residual volume and well-being, and to determine adverse events. This was a single centre, single-blinded, parallel-group, sex-stratified randomized controlled trial. Participants were randomized either to carbohydrate-rich whey protein beverage group (Resource^®^, Nestle Health Science) or control group (250 ml plain water) given pre-gastroscopy. Gastric contents were aspirated into a suction reservoir bottle to determine the gastric residual volume (GRV). Visual analogue scale (VAS) of well-being (anxiety, hunger, thirst, tiredness, and weakness) was compared before and after the intervention. Adverse events were also evaluated post-intervention. Of 369 screened, 78 participants (36 males, mean age 49 ± 14.3 years) were randomized. Compared with the control group, carbohydrate beverage was associated with significantly higher GRV (p < 0.001). Anxiety was less after intervention with carbohydrate beverage (p = 0.016), and after adjustment for confounders, fewer participants also experienced hunger (p = 0.043) and thirst (p = 0.021). No serious adverse events were reported with both interventions. Commercial carbohydrate-rich whey protein beverage is associated with higher gastric residual volume, better well-being and safe.

**Trial registration** Clinicaltrial.gov. Identifier: NCT03948594, Date of registration: 14/05/2019.

## Introduction

A gastric content > 25 ml (> 0.4 ml/kg) and pH < 2.5 are the basis of intentionally prolonged fasting for all patients undergoing surgery^1^. However, the practice of enhanced recovery after surgery (ERAS) is a paradigm shift. First introduced by Professor Henrik Kehlet in the 1990s, ERAS aimed to modify physiological and psychological responses to hasten postoperative recovery^2,3^. Carbohydrate loading pre-surgery is one of the ERAS measure to improve patient outcome^4^, by shifting fasting state into fed state to reduce insulin resistance^5^.

Most commercial carbohydrate-rich beverages contain polymer (maltodextrin) at low osmolality which improve gastric emptying rate, and reduce postoperative nausea and vomiting^6,7^. Often 50 g of carbohydrate is adequate to stimulate insulin release, and reduce insulin resistance^8^. Carbohydrate loading has been to shown to improve patient’s well-being post-operatively including thirst, hunger, anxiety, and the perception of pain^7,9,10^.

While the benefits and safety of carbohydrate loading have been demonstrated based on previous studies^1,10–12^ however it is unclear if commercial carbohydrate beverage provides similar benefits pre-gastroscopy and if also safe post-procedure especially the risk of pulmonary aspiration. Our study aimed to compare the effects of Resource^®^ (Nestle Health Science, Malaysia), a commercial carbohydrate-rich whey protein beverage, vs. plain water on gastric residual volume and the patient’s well-being (hunger, thirst, weakness, tiredness, anxiety), and to evaluate for adverse events post-intervention.

## Methodology

### Study design and population

Study design was randomized (1:1), placebo-controlled, single-blinded, parallel group, with sex-stratified sampling in a single center. Inclusion criteria were participants aged over 18 years old, scheduled for elective gastroscopy, and exclusion criteria were participants with symptoms suggestive of gastric outlet obstructions, planned for colonoscopy at the same setting, and incomplete gastroscopy.

Sample size was calculated using the two means formula for the gastric residual volume, and paired difference formula for the patients’ well-being. For objective 1, sample size was calculated using the comparing two means formula. The ratio between group A and group B was set as 1 with standard deviation 18.46 based on previous study^13^. Mean difference was set as 12.5. Hence, the sample size was 35 in each arm. For objective 2, the sample size was calculated using the comparing paired difference formula. The expected standard deviation of paired differences was set as 2 times the expected mean of the paired differences. Hence, the sample size was 34 in each arm. The type I error was set at 5% (two-tailed), the type 2 error was 20% (to achieve 80% power), and the dropout rate was set at 10%. The estimated sample size was 78 subjects with 39 subjects in each arm.

Randomization was performed using stratified permuted blocks, a computer-generated 1:1 allocation sequence stratified by sex. Random block sizes of 6 was used. The sealed enveloped method was used to implement the allocation sequence, and results were concealed from the primary investigator.

### Study protocol

Patients scheduled for an elective gastroscopy were first briefed on the study protocol and recruited after informed consent. The recruitment period was 12 months, from April 2019 to March 2020. Participants were randomized into either clear water (group A) or carbohydrate beverage group (group B). Participants in group A were given 250 ml of drinking water while group B was given one pack of Resource^®^ (Nestle Health Science, Malaysia) beverage (237 mls with 53.6 g of carbohydrate and 9 g of whey protein) mixed in water at the same volume of 250 ml. They were instructed to consume the drink within 10 min upon serving. Gastroscopy procedure would be performed after 2 h of serving, and in our unit, the test was performed under oral local anaesthesia (10% lidocaine). After procedure, the participants were separated and prohibited from interactions with other study participants and endoscopists.

**Figure 1:**
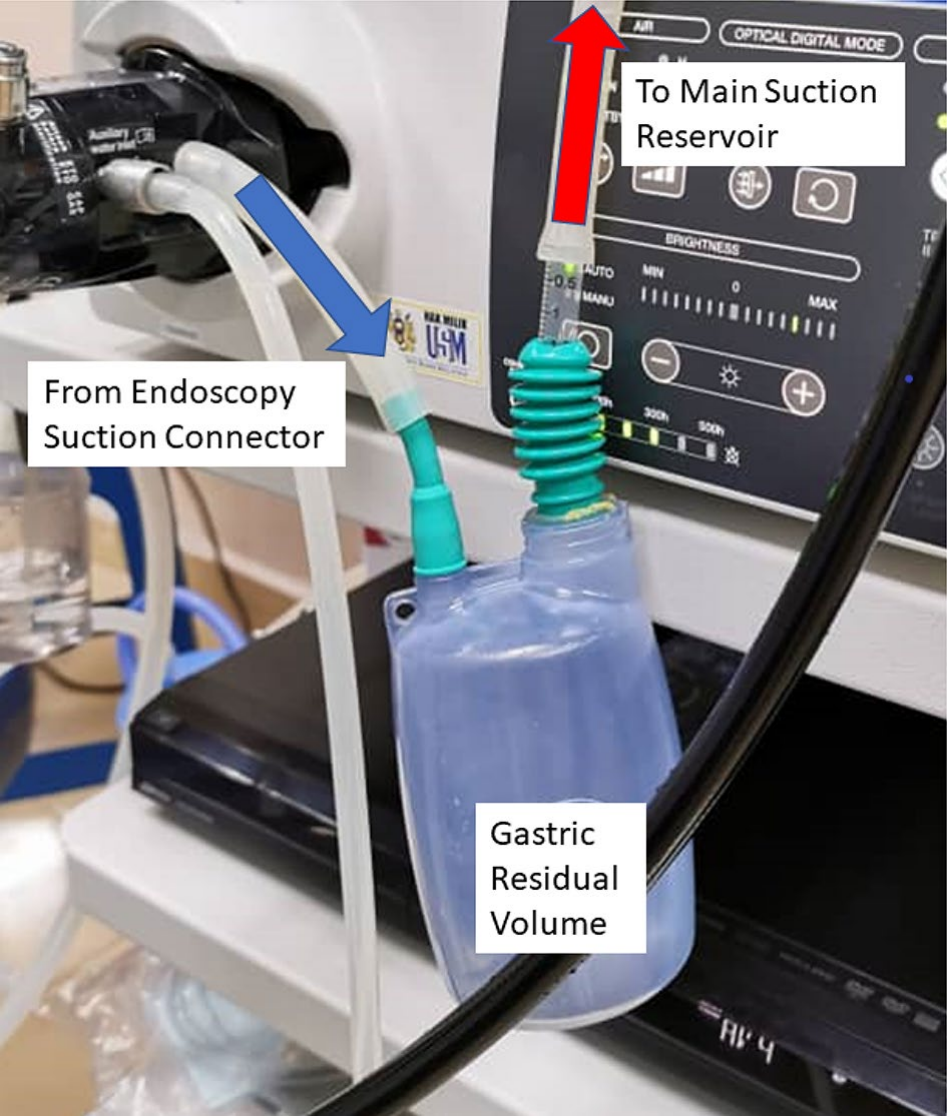
The suction reservoir bottle for GRV measurement.

In order to minimize effect on pre-measurement GRV, the endoscopists were asked not to use any high volume water flushing, but they were allowed limited lens-flushing if required. After GRV had been documented, endoscopists were allowed to resume normal water-jet and lens flushing for proper diagnostic examination. All the visible fluid residual in the stomach was aspirated via direct visualization using the gastroscope. Then, the fluid was collected into a suction reservoir bottle (Fig. 1) to record the GRV.

### Data analysis

Data was analyzed using SPSS version 26 (SPSS Inc., Chicago, IL, USA). Numerical data were presented as means (standard deviation, SD) or median (interquartile range, IQR) depending on distribution. For comparison of gastric residual volume between arm A and B, the independent T-test was used. To compare participants well-being (hunger, thirst, anxiety, tiredness and weakness) between groups, the repeated measure ANCOVA test was used.

### Ethics approval, trial registration and the CONSORT statement

Approval was obtained from the Human Research Ethics Committee of Universiti Sains Malaysia (USM/JEPeM/19010082 on 15/04/2019) that compliance with the Declaration of Helsinki, International Conference on Harmonization (ICH) Guidelines, good clinical practice (GCP) standards, Council for International Organizations of Medical Sciences (CIOMS) Guidelines, World Research and Surveying and Evaluating Ethical Review Practices, EC/IRB standard operating procedures (SOPs), and local regulations and standards in ethical review. This study was registered at the clinicaltrial.gov (NCT03948594 on 14/05/2019). Study protocol was performed according to the CONSORT statement and checklist.

## Results

**Figure 2:**
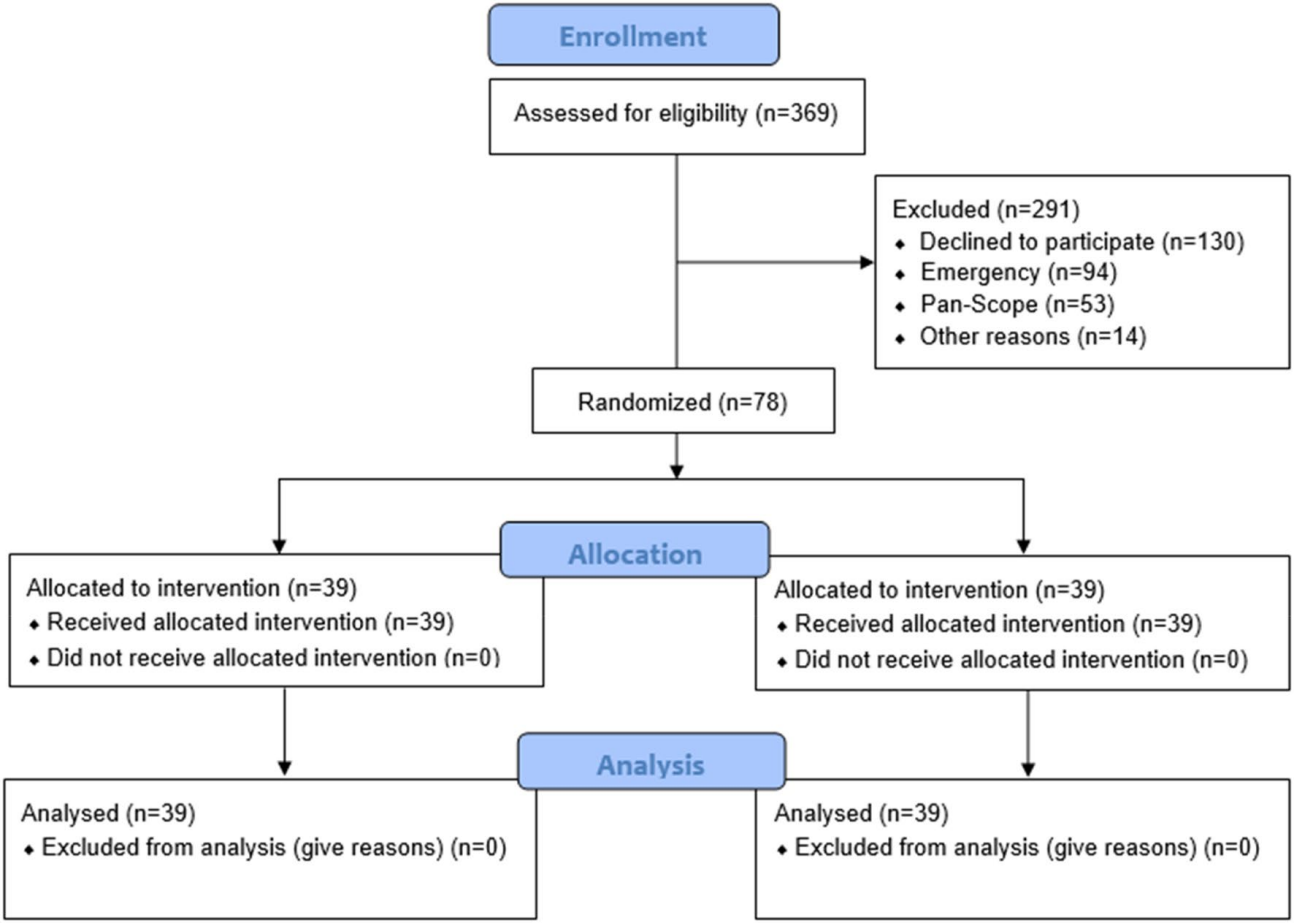
CONSORT flow of the study.

Of 369 patients undergoing gastroscopy, 291 patients were excluded (130 patients refused to consent, 94 were emergency gastroscopy, 53 also underwent colonoscopy and 14 patients presented as dysphagia and underwent oesophageal dilatation) and 78 were eventually recruited (Fig. 2). There were no significant differences between groups in sex, age, BMI distribution, time interval from drinks to gastroscopy, and findings during gastroscopy (Table 1).

**Table 1:** Demography and gastroscopy findings.

Variable	Frequency, n (%)		p-value
	Group A	Group B	
**Gender**			
Male	18 (46.2)	18 (46.2)	1.000^a^
Female	21 (53.8)	21 (53.8)	
Age (years)	49 (14.3)	49 (14.3)	0.436^b^
BMI (kg/m^2^)	26.49 (8.02)*	26.84 (10.29)*	0.513^c^
Interval from serving to gastroscopy (min)	131 (10.7)	132 (12.6)	0.714^b^
**Gastroscopy findings**			
Oesophagitis	6 (15.4)	8 (20.5)	0.555^a^
Gastritis	30 (76.9)	29 (74.4)	0.792^a^
Hiatus hernia	11 (28.2)	13 (33.3)	0.624^a^

*Median (IQR).

^a^Chi-square Test.

^b^Independent t-test.

^c^Mann-Whitney U test.

**Figure 3:**
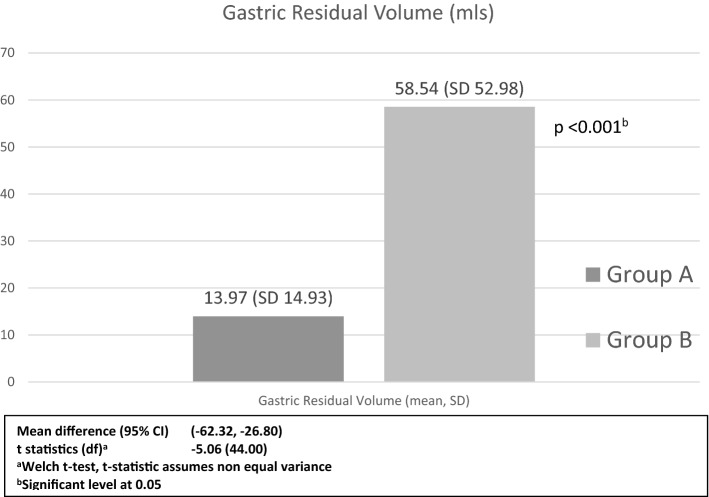
Comparison of gastric residual volume.

The mean GRV was significantly higher in group B 58.54 (52.98) ml vs. group A 13.97 (14.93) ml (p < 0.001) (Fig. 3). There were 77% (n = 30) participants in group B with GRV > 25 ml, and 26% (n = 10) with GRV ≥ 100 ml in comparison to 10% (n = 4) with GRV > 25 ml in group A. There was more thirst pre-intervention between the two groups (p = 0.043) otherwise other variables were not significantly different. Only anxiety was significantly reduced post-intervention between the two groups (p = 0.016) (Table 2). After adjustment for age, gender, BMI and time interval from drinks to gastroscopy, the VAS scores were different between groups for hunger (p = 0.043) and thirst (p = 0.021) but not statistically significant when compared between pre and post consumption (Table 3).

**Table 2:** Unadjusted mean for VAS measurement for participants’ well-being.

Variables	Group A (n = 39)	Group B (n = 39)	p-value
**Hunger**			
Pre (mean) (SD)	2.74 (2.15)	3.09 (2.06)	0.470
Post (mean) (SD)	3.19 (2.30)	2.44 (2.17)	0.145
**Thirst**			
Pre (mean) (SD)	2.70 (2.12)	3.76 (2.44)	0.043
Post (mean) (SD)	2.84 (2.36)	2.50 (2.08)	0.501
**Anxiety**			
Pre (mean) (SD)	2.96 (2.75)	2.38 (2.49)	0.334
Post (mean) (SD)	3.18 (2.30)	1.95 (2.09)	0.016
**Weakness**			
Pre (mean) (SD)	2.28 (2.02)	2.39 (2.70)	0.839
Post (mean) (SD)	2.42 (2.05)	2.06 (2.32)	0.474
**Tiredness**			
Pre (mean) (SD)	2.33 (2.50)	2.29 (2.44)	0.942
Post (mean) (SD)	2.40 (2.04)	2.05 (2.36)	0.481

**Table 3:** Adjusted mean and mean differences between groups A and B using repeated measure ANCOVA.

	Adj. Mean (SE)^a^	Adj. mean diff. (95% CI)^b^	F-stat	df	p-value
**Hunger**					
Pre (time 1)	2.93 (0.25)				
Group A	2.78 (0.35)	0.30 (− 0.68, 1.29)^§^	0.37	1, 71	0.544
Group B	3.08 (0.35)				
Post (time 2)	2.83 (0.26)				
Group A	3.20 (0.37)	− 0.74 (− 1.79, 0.31)	1.96	1, 71	0.166
Group B	2.47 (0.37)				
**Thirst**					
Pre (time 1)	3.24 (0.27)				
Group A	2.75 (0.37)	0.98 (− 0.08, 2.048)^¥^	3.39	1, 71	0.070
Group B	3.73 (0.37)				
Post (time 2)	2.69 (0.26)				
Group A	2.86 (0.37)	− 0.34 (− 1.38, 0.70)	0.43	1, 71	0.512
Group B	2.52 (0.37)				
**Anxiety**					
Group A	3.06 (0.36)	− 0.925 (− 1.95, 0.098)^¤^	3.247	1, 71	0.076
Group B	2.13 (0.36)				
**Tiredness**					
Group A	2.35 (0.34)	− 0.18 (− 1.14, 0.78)^†^	0.137	1, 71	0.712
Group B	2.17 (0.34)				
**Weakness**					
Group A	2.33 (0.34)	− 0.86 (− 1.05, 0.88)^₤^	0.031	1, 71	0.860
Group B	2.24 (0.34)				

^a^Adjusted means using RM ANCOVA with pre-post, age, gender, BMI and duration between scope.

^b^Bonferroni adjustment for 95% CI for difference.

^§^No significant difference over time (pre & post consumption) [F (df) = 0.30 (1, 71), p = 0.585], however there was significant interaction between time (pre & post consumption) and group [F (df) = 4.25 (1, 71), p = 0.043].

^¥^No significant difference over time (pre & post consumption) [F (df) = 0.01 (1, 71), p = 0.906], however there was significant interaction between time (pre & post consumption) and group [F (df) = 5.56 (1, 71), p = 0.021].

^¤^No significant difference over time [F (df) = 0.04 (1, 71), p = 0.840] and no significant interaction between time and group [F (df) = 2.10 (1, 71), p = 0.151].

^†^No significant difference over time [F (df) = 1.38 (1, 71), p = 0.243] and no significant interaction between time and group [F (df) = 0.31 (1, 71), p = 0.578].

^₤^No significant difference over time [F (df) = 0.27 (1, 71), p = 0.606] and no significant interaction between time and group [F (df) = 1.25 (1, 71), p = 0.267].

Regarding adverse events, there were no reported choking or aspiration during the procedure, and or abandoned gastroscopy due to overt gastric residue volume and or readmission of these patients from aspiration pneumonia.

## Discussion

The current study shows that compared to plain water, the commercial carbohydrate beverage, Resource^®^ given pre-gastroscopy resulted in higher gastric residual volume (GRV) post-gastroscopy. Our study demonstrated a higher GRV after carbohydrate load, different from a previously reported study^10^. The gastric residual volume was measured via direct aspiration during gastroscopy; a more accurate measurement than other indirect measurements for example dilutional or aspiration from the nasogastric tube^1,10^. However, this method might overestimate gastric residual volume. None of participants were obese and BMIs were similar at baseline in both groups, however obesity was not found to be a confounding factor in a study^14^, although insulin resistance was not investigated in the current study.

The carbohydrate beverage used in this study was Resource^®^ (Nestle Health Science, Malaysia), consisting of 53.6 g of carbohydrate in the form of maltodextrin. To achieve adequate induction of insulin release and shift body metabolism from fasting to a fed state, 50 g of carbohydrate is sufficient^8^. Polymer and low osmolality carbohydrates including maltodextrin have been shown to reduce gastric emptying time^6^. However, a study comparing the gastric emptying rate of water and protein drinks in older men and women concluded that higher protein content drinks significantly reduced the gastric emptying rate^15^. With Resource^®^, the 9 g of whey protein might contribute to higher GRV by reducing gastric emptying. In addition, higher osmolality beverage also delay gastric emptying^16,17^, and with an osmolality of 770 mOsm/kg H_2_O in Resource^®^, this could be another contributing factor to a higher GRV.

Prolonged fasting imposes stress on patients both physiologically and psychologically. Carbohydrate loading provides energy and fluid replenishment while reducing anxiety and lethargy related to fasting^10^. This explains why only anxiety, but not other variables of well-being were significantly reduced after carbohydrate beverage. In addition, there was less hunger and thirst with carbohydrate beverage after adjustment for BMI, age, and time interval for drinks to procedure. Other studies have also reported that carbohydrate loading improved hunger and thirst^10,18,19^.

There were no reported adverse events during the study. There was no pulmonary aspiration, failed or incomplete endoscopy secondary to overt GRV or choking. Although group B had higher GRV, there was no evidence of choking or aspiration. If carbohydrate-rich whey protein beverage is used as carbohydrate loading agent for general anaesthesia, then it should be coupled with anti-aspiration prophylaxis such as cricoid pressure, acid-suppression therapy and prokinetic agent before or during induction. However, all gastroscopy tests in our study were performed under oral local anaesthesia, hence, the risk of choking and pulmonary aspiration would be considered minimal. The absence of adverse events in our study provided further safety assurance of carbohydrate-rich beverage and clear fluid for up to 2 h prior to gastroscopy^20^.

There are study limitations. Participants with comorbidities such as diabetes mellitus, chronic kidney disease, gastro-oesophageal reflux disease and acute gastritis were not excluded, and some of these conditions might cause delayed gastric emptying. Although we did not collect data on these diseases, the limited nature of our intervention would not have affected activity these diseases even if these diseases were present. An ideal carbohydrate load should be free from protein or hyperosmolar additives which may affect gastric emptying however such ideal beverage is not commercially available.

In conclusion, carbohydrate-rich whey protein beverage results in higher GRV post-procedure, reduces anxiety post-procedure, and improves hunger and thirst after adjustment for confounding factors. The beverage given pre-procedure does not result in adverse events post-procedure.

## Data Availability

The dataset generated and/or analysed during this current study are not publicly available as the raw dataset has incorporated with participants identifiable information. Data are however available from the corresponding author on reasonable request.
